# The Prognostic Role of Volumetric MRI Evaluation in the Surgical Treatment of Glioblastoma

**DOI:** 10.3390/jcm13030849

**Published:** 2024-02-01

**Authors:** Denis Aiudi, Alessio Iacoangeli, Mauro Dobran, Gabriele Polonara, Mario Chiapponi, Andrea Mattioli, Maurizio Gladi, Maurizio Iacoangeli

**Affiliations:** 1 Department of Neurosurgery, Università Politecnica delle Marche, Azienda Ospedaliero Universitaria delle Marche, 60121 Ancona, Italy; alessio.iacoangeli95@gmail.com (A.I.); dobran@libero.it (M.D.); mario.chiapponi.med@gmail.com (M.C.); andrea.mattioli.92@gmail.com (A.M.); mauriziogladi@gmail.com (M.G.); neurotra@gmail.com (M.I.); 2 Department of Neuroradiology, Università Politecnica delle Marche, Azienda Ospedaliero Universitaria delle Marche, 60121 Ancona, Italy; gabriele.polonara@ospedaliriuniti.marche.it

**Keywords:** FLAIR infiltration, brain tumors, extent of surgical resection, glioblastoma, overall survival, progression-free survival, pseudocapsule, neuro-oncology, tumor volume

## Abstract

**Background**: Glioblastoma is the most common primary brain neoplasm in adults, with a poor prognosis despite a constant effort to improve patient survival. Some neuroradiological volumetric parameters seem to play a predictive role in overall survival (OS) and progression-free survival (PFS). The aim of this study was to analyze the impact of the volumetric areas of contrast-enhancing tumors and perineoplastic edema on the survival of patients treated for glioblastoma. **Methods**: A series of 87 patients who underwent surgery was retrospectively analyzed; OS and PFS were considered the end points of the study. For each patient, a multidisciplinary revision was conducted in collaboration with the Neuroradiology and Neuro-Oncology Board. Manual and semiautomatic measurements were adopted to perform the radiological evaluation, and the following quantitative parameters were retrospectively analyzed: contrast enhancement preoperative tumor volume (CE-PTV), contrast enhancement postoperative tumor volume (CE-RTV), edema/infiltration preoperative volume (T2/FLAIR-PV), edema/infiltration postoperative volume (T2/FLAIR-RV), necrosis volume inside the tumor (NV), and total tumor volume including necrosis (TV). **Results**: The median OS value was 9 months, and the median PFS value was 4 months; the mean values were 12.3 and 6.9 months, respectively. Multivariate analysis showed that the OS-related factors were adjuvant chemoradiotherapy (*p* < 0.0001), CE-PTV < 15 cm^3^ (*p* = 0.03), surgical resection > 95% (*p* = 0.004), and the presence of a “pseudocapsulated” radiological morphology (*p* = 0.04). **Conclusions**: Maximal safe resection is one of the most relevant predictive factors for patient survival. Semiautomatic preoperative MRI evaluation could play a key role in prognostically categorizing these tumors.

## 1. Introduction

Glioblastoma (GB) is the most common primary brain neoplasm in adults and the most common malignancy of the CNS (approximately 49% of malignant brain tumors are glioblastomas) [1], It is described by the WHO as grade 4 according to the most recent updates to the WHO classification (2021) [2,3].

Age, sex, and race/ethnicity influence the incidence rate, which exponentially increases beyond 40 years of age. The mean age of diagnosis is 65 years, and it peaks between 75 and 84 years. GB is more common in males and Caucasians compared to African-American patients [4].

Adult-type diffuse gliomas now consist of only three categories: astrocytoma, IDH-mutant; oligodendroglioma, IDH-mutant and 1p/19-codeleted; and glioblastoma, IDH-wildtype. Thus, astrocytic tumors are grouped as those with and without IDH mutations; those without IDH mutations (wildtype) are named glioblastomas IDH-wildtype. The term “glioblastoma multiforme” should not be used [1,2].

Despite decades of advances in surgery and discoveries in molecular research, encouraging outcomes are not typically observed; patients diagnosed with this tumor generally have a dismal prognosis and poor quality of life as the disease progresses. The median survival time has been reported to be less than 15 months on average. Survival longer than 3–5 years has been reported for approximately 0.5% of GB patients [5]. 

These data have led to an increasing number of studies focused on acquiring knowledge about GB prognostic factors. According to the literature, the most relevant prognostic factors are age, sex, Karnofsky Performance Status (KPS), surgical resection rate, adjuvant therapies performed, and tumor molecular biology [6]. This last characteristic has grown in importance because several genetic mutations have been shown to have a prognostic role, such as MGMT promoter methylation, loss of 10q heterozygosity, miRNA dysregulation, EGFR mutation, PTEN mutation, P53 mutation, and especially IDH1 mutation. 

Positive GB prognostic elements:

MGMT promoter methylation: Methylation of the MGMT (O6-methylguanine-DNA methyltransferase) promoter is associated with an improved response to temozolomide chemotherapy, leading to a more favorable prognosis in glioblastoma patients.

ATRX mutations: ATRX mutations, particularly in the context of the IDH-mutant, 1p/19q non-codeleted subtype, are generally associated with a more favorable prognosis and longer overall survival rates.

IDH1 R132H and IDH2 R173 mutations: In the rare instances of glioblastoma harboring these specific IDH mutations, patients tend to have a better prognosis compared to IDH wildtype glioblastomas. However, it is important to note that these mutations are relatively rare in glioblastoma.

Negative GB prognostic elements:

EGFR amplification: Amplification of the EGFR (epidermal growth factor receptor) gene is associated with increased tumor aggressiveness and a poorer prognosis in glioblastoma patients.

TERTp mutations: Mutations in the TERT (telomerase reverse transcriptase) promoter are often associated with increased telomerase activity and contribute to the aggressiveness of glioblastoma, resulting in a poorer prognosis.

Gain of Chr.7 and loss of Chr.10: Chromosomal alterations involving the gain of chromosome 7 and the loss of chromosome 10 are commonly observed in glioblastoma and are associated with more aggressive tumor behavior and a worse prognosis [1].

Recent studies have also shown increasing interest in some neuroradiological parameters, evaluated both prior and after surgery, that seem to play a predictive role in overall survival (OS) and progression-free survival (PFS) [7,8,9,10].

Glioblastomas are typically large tumors at diagnosis. They often have thick, irregularly enhancing margins and a central necrotic core, which may also have a hemorrhagic component. They are characterized by their ability to invade surrounding parenchyma and are usually surrounded by vasogenic-type edema, which, in fact, usually contains infiltration by neoplastic cells, making curative resection difficult.

Contrast-enhanced brain magnetic resonance imaging is the gold standard for diagnosis and presurgical planning. T1-weighted (T1) and T2-weighted/fluid-attenuated inversion recovery (T2/FLAIR) sequences are commonly used in the study of glioblastoma.

T1-weighted images provide good anatomical detail and are excellent for visualizing the brain’s anatomy, and GB typically appear hypointense (dark) due to their high cellularity and increased protein content, making them distinguishable from surrounding normal brain tissues. Enhancement patterns on T1 postcontrast images are often present and are typically peripheral and irregular with nodular components. They are usually indicative of increased vascularity and blood–brain barrier disruption surrounding a necrotic core, which may also have a hemorrhagic component.

T2-weighted images are sensitive to variations in water content and are useful for highlighting vasogenic-type edema, which usually contains infiltration by neoplastic cells and typically appears hyperintense (bright) in GB. FLAIR sequences suppress cerebrospinal fluid (CSF) signals, enhancing the visibility of abnormalities near CSF-filled spaces and making it easier to identify tumor borders.

The extent of edema seen on T2/FLAIR images can provide information about the tumor’s infiltrative nature and its impact on the surrounding brain tissues. The absence of a T2/FLAIR mismatch may also help with differential diagnosis.

The aim of our study was to analyze clinical, radiological, and histologic characteristics as predictive factors for OS and PFS in patients affected by GB who underwent surgery and were monitored at our institute; in particular, the impact of the volumetric areas of contrast-enhancing tumor and perineoplastic edema on the outcome of patients was analyzed.

## 2. Materials and Methods

A series of 87 patients diagnosed with GB (glioblastoma, IDH-wildtype, CNS WHO grade 4) who underwent surgery at our institution between 2020 and 2022 was retrospectively analyzed.

Overall survival (OS, defined as the time from first surgery until death) and progression-free survival (PFS, defined as the time from first surgery and the radiological evidence of disease relapse/progression on MRI) were considered the end points of the study. For each patient, demographic, clinical, radiological, and histological characteristics were studied as predictive factors, and a multidisciplinary revision of medical records was conducted in collaboration with the Neuroradiology and Neuro-Oncology Board.

Manual and semiautomatic measurements were adopted to perform the radiological evaluation, and the following quantitative parameters were retrospectively analyzed: contrast enhancement preoperative tumor volume (CE-PTV), contrast enhancement postoperative tumor volume (CE-RTV), edema/infiltration preoperative volume (T2/FLAIR-PV), edema/infiltration postoperative volume (T2/FLAIR-RV), necrosis volume inside the tumor (NV), and total tumor volume including necrosis (TV). Quantitative volumetric assessment was carried out using the Advantage Workstation Server 3.2 (AW Server 3.2, General Electric^®^, 2009–2015, Boston, MA, USA).

A presurgery MRI was available for all patients; 37 (42.5%) of them also underwent a postoperative MRI within the first 48 h after surgery. All exams were performed on 1.5 T scanners.

**Figure 1:**
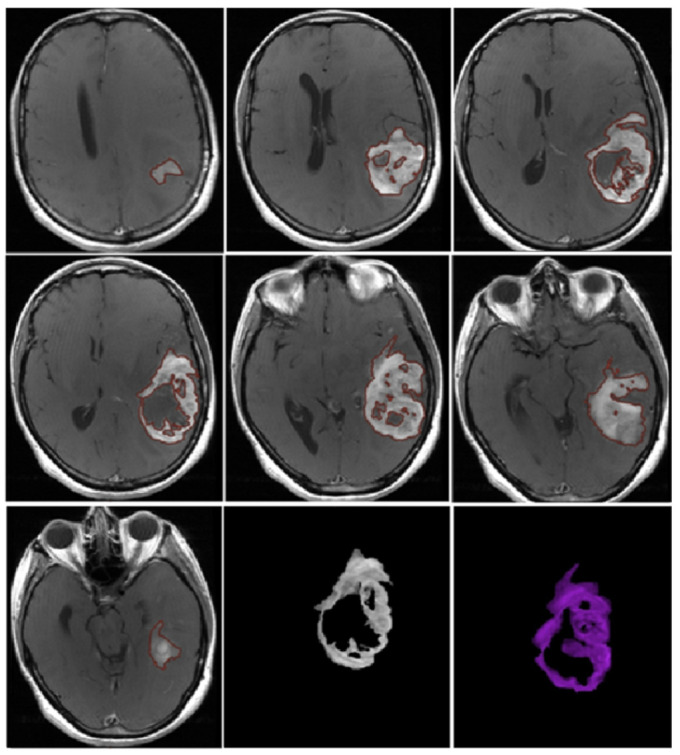
CE-PTV, contrast-enhanced T1 weighted (CE-T1w) images by contouring manually enhanced tumor areas on every single axial slice, excluding necrosis.

**Figure 2:**
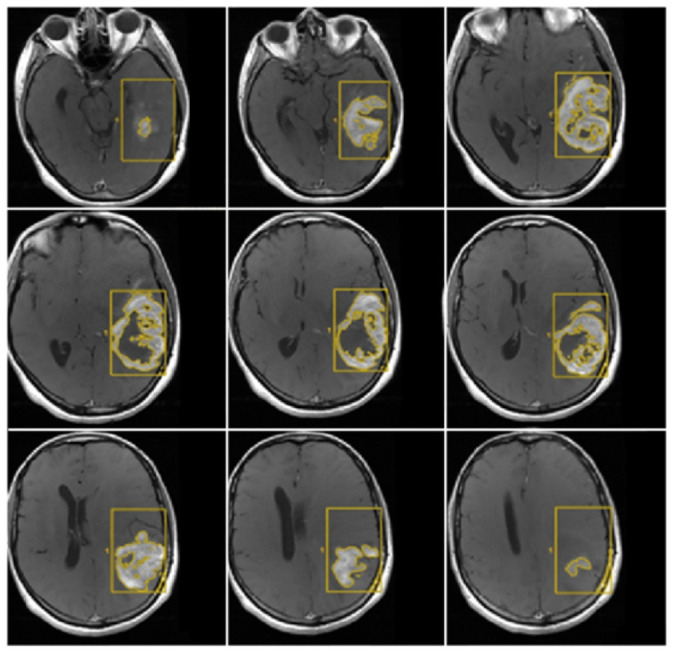
CE-PTV, contrast-enhanced T1 weighted (CE-T1w) images performed with the semiautomatic method using the specific tool of the AW Server 3.2. The yellow box represents the area selected by the radiologist that the software analyzes (semi-automatic method).

CE-PTV was evaluated on 2D axial contrast-enhanced T1 weighted (CE-T1w) images (slice thickness: 5 mm; slice spacing: 5.5–6 mm) by contouring manually enhanced tumor areas on every single axial slice, excluding necrosis; the same analysis was subsequently performed with the semiautomatic method using the specific tool of the AW Server 3.2 (Figure 1 and Figure 2).

**Figure 3:**
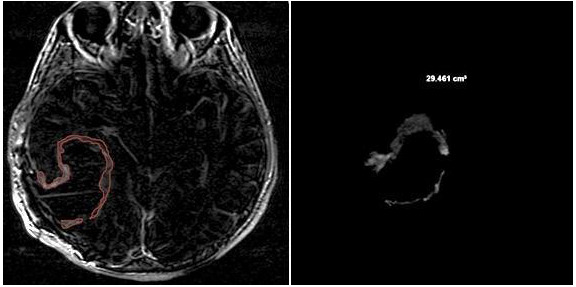
Contrast enhancement postoperative tumor volume (CE-RTV) manual evaluation, achieved with subtraction imaging technique to minimize errors due to the spontaneous hyperintensity of degradation products of hemoglobin.

**Figure 4:**
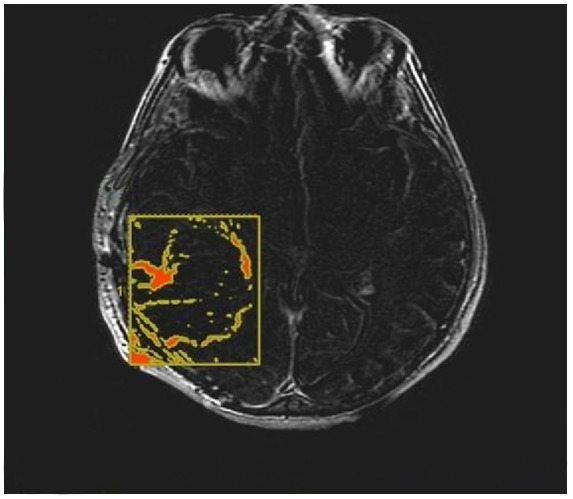
Contrast enhancement postoperative tumor volume (CE-RTV) semiautomatic evaluation, assessed on 2D axial CE-T1w images (slice thickness: 5 mm; slice spacing: 5.5–6 mm), achieved with the semiautomatic subtraction imaging technique.

CE-RTV was assessed on 2D axial CE-T1w images (slice thickness: 5 mm; slice spacing: 5.5–6 mm) with the subtraction imaging technique to minimize errors due to the spontaneous hyperintensity of degradation products of hemoglobin or those related to the presence of hemostatic/chemotherapeutic agents in the surgical area. As for CE-PTV, the analysis was performed both manually and semiautomatically (Figure 3 and Figure 4).

**Figure 5:**
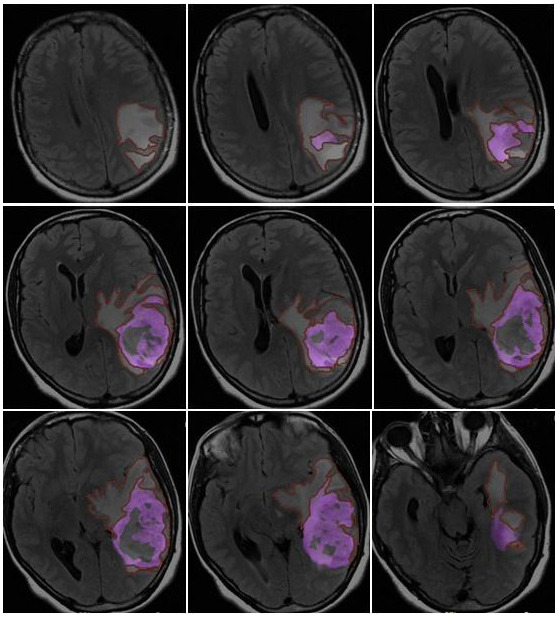
Edema/infiltration preoperative volume (T2/FLAIR-PV), evaluated manually on axial hybrid sequences resulting from FLAIR (slice thickness: 5 mm; slice spacing: 5.5 mm) and CE-T1w in order to exclusively measure the edema/infiltration component, excluding the tumor (enhancing mass) previously assessed with CE-PTV and CE-RTV measurements.

**Figure 6:**
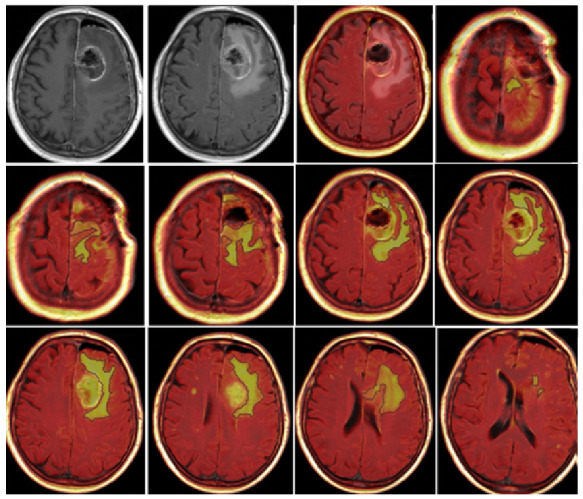
Edema/infiltration postoperative volume (T2/FLAIR-RV), evaluated manually on axial hybrid sequences resulting from FLAIR and CE-T1w fusion in order to exclusively measure the edema/infiltration component, excluding the tumor (enhancing mass).

T2/FLAIR-PV and T2/FLAIR-RV were both evaluated manually on axial hybrid sequences resulting from FLAIR (slice thickness: 5 mm; slice spacing: 5.5 mm) and CE-T1w (slice thickness: 5 mm; slice spacing: 5.5–6 mm) fusion in order to exclusively measure the edema/infiltration component, excluding the tumor (enhancing mass) previously assessed with CE-PTV and CE-RTV measurements (Figure 5 and Figure 6).

**Figure 7:**
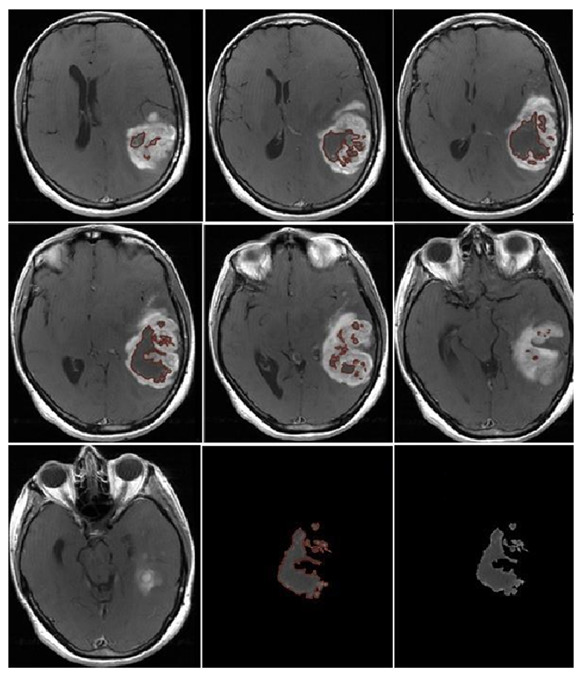
Necrosis volume inside the tumor, evaluated manually on preoperative 2D axial CE-T1w images, including only the necrotic area inside the tumor.

NV was evaluated manually on preoperative 2D axial CE-T1w images, including only the necrotic area inside the tumor (Figure 7).

TV was calculated as the sum of NV and CE-PTV, both assessed on 2D axial CE-T1w images (slice thickness: 5 mm; slice spacing: 5.5–6 mm).

In the case of tumor localization near eloquent brain areas, the extent of surgical resection was modulated based on neurophysiological monitoring techniques such as sensorimotor evoked potentials and electrocorticography.

**Figure 8:**
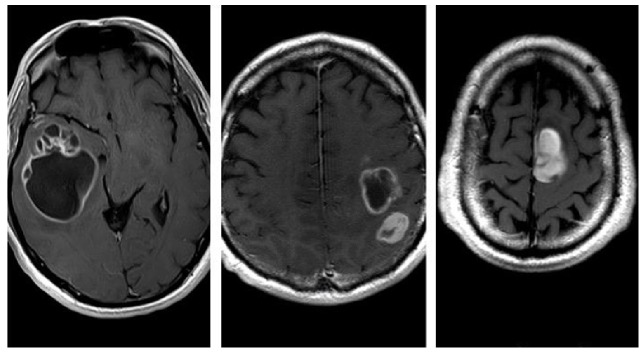
Thin, with enhancing wall thickness < 3 mm (**left**); thin-nodular, with enhancing wall focal thickenings > 3 mm (**center**); and nodular, with solid appearance predominant and intratumoral necrosis absent or less than 1.5 cm³ (**right**).

Furthermore, we investigated the following relevant qualitative characteristics of GB: tumor localization (the lobe containing the enhancing mass or, in case of radiological multifocality, the lobe corresponding to the main tumor mass was considered as the tumor site); eloquent area involvement (defined as neoplastic infiltration of the cortex or iuxta-cortical white matter of eloquent areas, such as motor, visual, Wernicke’s area, or Broca’s area); and radiological appearance (distinguished by three different patterns based on the enhancing wall thickness on CE-T1w sequences: thin, with enhancing wall thickness < 3 mm; thin-nodular, with enhancing wall showing focal thickening > 3 mm; and nodular, with solid appearance predominant and intratumoral necrosis absent or less than 1.5 cm^3^) (Figure 8). Similarly, we identified two morphological categories: “pseudocapsulated” and non-pseudo-capsulated masses, depending on the macroscopic appearance of a pseudocleavage plane at the time of neurosurgery. Furthermore, we analyzed the presence of ependymal involvement (defined as visible signal alteration on FLAIR images or tumor mass joining the ependymal interface) and focal or multifocal disease (focal if only a single mass was observed; multifocal if multiple tumor foci were visible, contiguous to FLAIR signal alterations or not, with no difference between the terms “multifocal” and “multicenter”).

Patients with incomplete data sets were not included in the study sample.

Statistical analysis was performed using the MedCalc 15.8 Portable software.

Univariate analysis was carried out using the Kaplan–Meier method, and patient subgroups were compared employing the log-rank test. Both univariate and multivariate analyses were based on the Cox proportional hazard regression stepwise method to identify predictive factors for OS and PFS.

A *p*-value < 0.05 was considered statistically significant.

All procedures performed in the study were conducted in accordance with the ethics standards given in the 1964 Declaration of Helsinki, as revised in 2013. The study protocol was approved by the Institutional Ethics Review Board at our institution. All participants provided written informed consent for their participation in the study, and patient consent was obtained for the purpose of the study with due care to maintain their privacy.

## 3. Results

Our sample included 87 patients; 45 (51.7%) were males and 42 (48.3%) were females, and the mean age was 67 years (range: 25–85 years). At the time of our study, 22 (25.3%) patients were still alive, and 65 (74.7%) were deceased.

All patients underwent neurosurgical intervention; 68 (78.1%) of them received adjuvant therapy as follows: 4 (4.6%) patients received only chemotherapy, 7 (8%) patients received only radiotherapy, and 57 (65.5%) received both chemo- and radiotherapy. A total of 19 (21.9%) patients did not receive any adjuvant treatment.

The median OS was 9 months, and the median PFS was 4 months; the mean values were 12.3 months for OS and 6.9 months for PFS.

The KPS was evaluated before and after surgery: 74 (85%) patients showed a preoperative KPS > 80 and 13 (15%) had a preoperative KPS < 80; two months after surgery, there were 49 (59%) patients with a postoperative KPS < 80, while there were 34 (41%) patients who had a postoperative KPS > 80. Four patients died within the first month after surgery.

### 3.1. Qualitative Analysis

- Localization: All tumors had a supratentorial localization; 31 (36%) were in the frontal lobe, 18 (21%) were in the parietal lobe, 36 (41%) in the temporal lobe, and 2 (2%) in the occipital lobe.
- Eloquent areas: 35 of the 87 lesions (40%) were in eloquent areas.
- Ependymal involvement: Ependymal involvement was observed in 52 (60%) patients; 35 (40%) lesions had no connection with the periventricular zone.
- Morphological appearance: We divided GB lesions into three categories based on the enhancing wall thickness: thin, <3 mm; thin-nodular, when the enhancing wall showed focal thickenings > 3 mm; and nodular, when solid appearance was predominant and intratumoral necrosis was absent or <1.5 cm^3^. A total of 11 (13%) masses showed a thin pattern, 51 (58%) showed a thin-nodular pattern, and 25 (29%) showed a nodular pattern.
- Multifocal disease: Multifocal disease was found in 20 (23.3%) patients (Table 1).

### 3.2. Quantitative Analysis

Median CE-PTV values obtained by manual and semiautomatic methods were 24.8 and 22.6 cm^3^, respectively. A good concordance value (R^2^: 0.86) between manual and semiautomatic measurements was observed; a greater dispersion rate was noticeable for volume values > 25 cm^3^.

We also calculated the percent deviation between manual and semiautomatic volumes, and the resulting deviation mean value was 4%, despite a mean squared deviation value of 34%. This result is probably due to the high values of the mean squared deviation corresponding to mass volumes > 40 cm^3^. Similar results were observed for the CE-RTV manual and semiautomatic volume correlation; the CE-RTV median values obtained by manual and semiautomatic methods were 5.8 and 6.8 cm^3^, respectively.

The TV median value was 37.7 cm^3^, and the surgical resection median value was 78%.

T2/FLAIR-PV and T2/FLAIR-RV were 59.2 and 42.3 cm^3^, respectively; the preoperative necrosis volume median value was 6.6 cm^3^.

### 3.3. Overall Survival—Univariate Analysis

The univariate analysis showed that adjuvant therapies (chemotherapy, radiotherapy, and chemoradiotherapy) and CE-RTV < 5.8 cm^3^ were the only variables connected with OS.

- Chemotherapy: The median OS values were 15 months for patients who received adjuvant chemotherapy (n = 61) and 3 months for patients who did not (n = 26); the difference between the two groups was statistically significant (*p* < 0.0001).
- Radiotherapy: The median OS values were 14 months for patients who received adjuvant radiotherapy (n = 64) and 3 months for patients who did not (n = 23); the difference between the two groups was statistically significant (*p* < 0.0001).
- Chemoradiotherapy: The median OS values were 16 months for patients who received chemoradiotherapy (n = 57), 6 months for patients who received radiotherapy alone (n = 7), and 5 months for patients who received chemotherapy alone (n = 4); the median OS value was 2 months for patients who did not receive any adjuvant treatment (n = 19). The difference was statistically significant (*p* < 0.0001).
- CE-RTV: The median OS value was 19 months for patients with CE-RTV < 5.8 cm^3^ and 9 months for patients with CE-RTV > 5.8 cm^3^. The difference was statistically significant (*p* < 0.004) (Table 2).

### 3.4. Overall Survival—Multivariate Analysis

Similar to univariate analysis, multivariate analysis showed that adjuvant chemo- and radiotherapy were OS-related prognostic factors (*p* < 0.0001). Furthermore, multivariate analysis proved that the only OS-related radiological prognostic factor was CE-PTV < 15 cm^3^ (*p* = 0.03).

### 3.5. Progression-Free Survival—Univariate Analysis

During follow-up, 70 patients showed disease relapse or progression; the median PFS value was 4 months, while the mean PFS value was 6.9 months.

Univariate analysis demonstrated that PFS-related variables were gender, adjuvant chemo- and radiotherapy, postoperative KPS, and CE-RTV.

- Gender: The median PFS value was 4 months for men and 5 months for women. The difference was statistically significant (*p* = 0.02).
- Chemotherapy: The median PFS value was 6 months for patients who underwent chemotherapy and 1 month for patients who did not. The difference was statistically significant (*p* < 0.0001).
- Radiotherapy: The median PFS value was 5 months for patients who received radiotherapy and 1 month for patients who did not. The difference was statistically significant (*p* < 0.0001).
- Postoperative KPS: The median PFS value was 3 months in patients with postoperative KPS < 80 and 7 months in patients with postoperative KPS > 80. The difference was statistically significant (*p* < 0.0001).
- CE-RTV: The median PFS value was 5 months in patients with CE-RTV < 5.8 cm^3^ and 4 months in patients with CE-RTV > 5.8 cm^3^. The difference was statistically significant (*p* = 0.04).
- Surgical resection: The median PFS value was 6 months in patients with a surgical resection percentage > 95% and 4 months if the surgical resection percentage was < 95%. The difference was statistically significant (*p* = 0.02).

### 3.6. Progression-Free Survival—Multivariate Analysis

PFS-related variables in multivariate analysis were adjuvant radiotherapy (*p* = 0.01), surgical resection percentage > 95% (*p* = 0.004), and the presence of “pseudocapsulated” morphologic gross appearance (*p* = 0.04).

The preliminary analysis, performed with the McNemar test for qualitative dichotomous nominal variables to find a correlation between “pseudocapsulated” appearance and other parameters, showed a strong match between the macroscopic presence of pseudocapsule and the nodular pattern observed in preoperative MRI (*p* < 0.0001) (Table 3).

All quantitative data for CE-PTV and CE-RTV evaluations are shown in Table 4.

## 4. Discussion

According to the most recent literature, OS median values in patients with GB ranges from 6 to 14 months [11], while the PFS median value is 6 months [12]. Our data (OS median value: 9 months; PFS median value: 4 months) seems to be consistent with this evidence. CE-PTV and TV median values of our sample were 24 and 39 cm^3^, respectively, compared to other studies such as Wangaryattawanich et al.’s series that reported a median CE-PTV = 21 cm^3^ and Ellingson et al.’s work with a median TV = 15 cm^3^; this difference may depend on the fact that larger masses were resected in our series, and this could explain our lower survival rate [13,14]. CE-PTV < 15 cm^3^ was an independent predictive factor for OS together with adjuvant radio and chemotherapy in multivariate analysis; especially when combined, it significantly increased the OS. Total tumor volume including necrosis (TV), with a 15 cm^3^ cut-off, represented a relevant independent prognostic factor in multivariate analysis, in accordance with the aforementioned studies. Additionally, these findings underscore the importance of accurate tumor volume assessment for optimizing treatment strategies and predicting outcomes in GB patients.

Chaichana et al. established thresholds for the extent of resection and residual volume that impact the survival and recurrence rates in patients with newly diagnosed intracranial glioblastoma. Our analysis confirmed these findings by analyzing postoperative enhancing mass volume (CE-RTV), resulting in a significance cut-off value of 5.8 cm^3^. This parameter emerged as an important independent prognostic factor in univariate analysis. Notably, patients with CE-RTV < 5.8 cm^3^ demonstrated superior OS and PFS values, emphasizing the pivotal role of surgical mass removal rates [15]. Univariate analysis concerning PFS revealed that factors such as female gender, adjuvant chemo- and radiotherapy, absence of postoperative neurological deficits, CE-RTV < 5.8 cm^3^, and surgical resection rate > 95% of TV significantly increased its median value. Multivariate analysis pointed towards adjuvant radiotherapy and surgical resection rate > 95% of TV as independent predictive prognostic factors. The absence of postoperative neurological deficits was a noteworthy parameter influencing PFS, leading to higher values in patients without deficiencies. This condition strongly correlated with Karnofsky Performance Status (KPS) values > 80, emphasizing the crucial role of KPS as a prognostic factor, a notion well-supported in the existing literature [16,17,18]. A reduction in KPS value postsurgery emerged as a negative prognostic factor. In our experience, a surgical resection rate > 95% proved to be a relevant independent prognostic factor for PFS in multivariate analysis, further affirming its significance alongside adjuvant chemo- and radiotherapy. These factors, which are well documented in the literature, played a decisive role in our series, significantly associating with both OS and PFS [6,19,20,21,22,23].

In our investigation, we identified an additional independent prognostic factor for progression-free survival (PFS): the presence of a morphological “pseudocapsulated” gross appearance in the tumoral mass. This phenomenon is characterized by a lesion displaying an apparent “pseudocapsule” at the superficial aspect, possibly attributed to regions of necrosis and hemorrhage, as suggested by Khadijeh Abdal et al. [24]. Our preliminary experience indicates that this more compact mass, with evidence of a pseudocleavage plan, is correlated with the nodular preoperative MRI pattern. To our knowledge, this specific association has not been widely documented in the existing literature. The identification of this aspect by the neurosurgeon, coupled with neuroradiological findings, may be related to the anatomopathological characterization.

Some tumors exhibit a type of “pseudoplane” surrounding the nodule, facilitating easier and more effective removal. Al-Holou et al. [8] demonstrated that circumferential perilesional resection of glioblastoma (GB) was linked to significantly higher rates of complete resection and lower rates of neurological complications, even for tumors in eloquent locations. Perilesional resection, when feasible, should be considered a preferred option.

Our analysis did not identify them as significant in our T2/FLAIR-PV and T2/FLAIR-RV cohorts as significant prognostic factors, even though some studies have suggested these volumetric parameters as important OS (T2/FLAIR-PV = 85 cm^3^) and PFS (T2/FLAIR-RV = 24.85 cm^3^) predictive factors. Grossman R. et al. [25] noticed that OS was related to CE-RTV assessed immediately after surgery and that a FLAIR alteration signal volume reduction of at least 46% of preoperative volume (or a postoperative FLAIR alteration signal volume < 19.3 cm^3^) evaluated 3 months after surgery represented a favorable prognostic factor for OS, suggesting that surgical resection beyond contrast-enhancing boundaries could represent a promising strategy to improve outcomes in GB patients. In relation to the amount of FLAIR abnormality removal (defined as the rate of resection of the infiltrative tumor component), Pessina et al. recorded a cut-off value for conditioning survival of 45% [9].

While these data necessitate correlation with main predictive factors such as KPS and adjuvant therapies, quantitative imaging emerges as a reliable and valuable tool in predicting overall and progression-free survival in glioblastoma patients undergoing surgery. Notably, the removal of the surrounding perinodular area stands out as a significant prognostic factor. Tumors exhibiting a nodular pattern, in our experience, correlate with enhanced surgical removal, leading to maximal safe asportation and an improved prognosis. This, however, needs to be carefully balanced with the goal of minimizing neurological deficits. Glioblastoma is not merely a surgical disease but a complex condition demanding multimodal treatment and multidisciplinary management. It remains crucial to acknowledge that increasing resection volume, at the cost of inducing new or permanent neurological deficits, may nullify the survival benefit conferred to patients. Therefore, a judicious approach is essential to optimizing both surgical outcomes and overall patient well-being.

## 5. Conclusions

In conclusion, despite the incorporation of diverse therapies, the prognosis for GB patients remains bleak. A radical, yet safe, maximal surgical resection still retains its role as a crucial predictive factor for patient survival. The integration of quantitative MRI volumetric imaging, particularly semiautomatic preoperative evaluation, emerges as pivotal in stratifying the prognostic categories of these tumors and shows the intricate interplay between surgical precision, imaging technologies, and overall patient outcomes. Therefore, advancing our understanding of these dynamics holds promise for refining treatment strategies and ultimately improving the challenging prognosis faced by GB patients.

## Figures and Tables

**Table 1:** Qualitative analysis.

Localization	n (%)	Morphology	n (%)
Frontal lobe	31 (36%)	Multifocal	20 (23.3%)
Parietal lobe	18 (21%)	Thin (d < 3 mm)	11 (13%)
Temporal lobe	36 (41%)	Thin-nodular (d > 3 mm)	51 (58%)
Occipital lobe	2 (2%)	Nodular	25 (29%)
Eloquent areas	35 (40%)	Pseudocapsulated	62 (71%)
Ependymal involvment	52 (60%)		

**Table 2:** Overall survival.

	Median OS Treated (n)	Median OS Not Treated (n)	*p*-Value
AC	15 (n = 61)	3 (n = 26)	<0.0001
ART	14 (n = 64)	3 (n = 23)	<0.0001
CRT	16 (n = 57)	2 (n = 19)	<0.0001
CT	5 (n = 4)		
RT	6 (n = 7)		
NT	2 (n = 19)		
CE-RTV	<5.8 cm^3^: 19	>5.8 cm^3^: 9	<0.004

OS, overall survival; AC, adjuvant chemotherapy; ART, adjuvant radiotherapy; CRT, chemoradiotherapy; CT, chemotherapy; RT, radiotherapy; NT, not treated.

**Table 3:** Progression-free survival.

	Median PFS (Months)		*p*-Value
Sex (M/F)	Male 4	Female 5	*p* = 0.02
Chemotherapy	With: 6 /	Without: 1	*p* < 0.0001
Radiotherapy	With: 5 /	Without: 1	*p* < 0.0001
Postoperative KPS	<80: 3	>80: 7	*p* < 0.0001
CE-RTV	<5.8 cm^3^: 5	>5.8 cm^3^: 4	*p* = 0.04
Surgical resection (%)	>95%: 6	>95%: 4	*p* = 0.02
Pseudocapsulated	with: 6	Without: 3	*p* < 0.0001

**Table 4:** Quantitative data for CE-PTV and CE-RTV evaluations.

Patient	Semiautomatic CE-PTV (cm^3^)	Manual CE-PTV (cm^3^)	CE-RTV (cm^3^)
1	34.89	31.14	
2	30.00	33.51	16.37
3	27.32	33.33	0.00
4	17.53	15.46	
5	12.21	5.24	
6	5.21	4.70	0.00
7	21.99	24.04	10.71
8	59.10	38.78	5.82
9	20.15	21.31	6.98
10	64.52	72.11	
11	11.83	10.58	10.12
12	49.81	49.72	
13	11.01	10.17	
14	20.87	17.97	
15	32.55	39.27	
16	46.11	42.10	
17	35.88	36.29	
18	23.65	39.15	
19	5.87	5.21	4.65
20	25.75	24.16	10.49
21	17.78	14.68	
22	20.43	21.26	
23	1.18	0.39	0.00
24	10.93	11.26	
25	16.06	14.84	0.00
26	20.11	18.65	13.28
27	25.89	25.40	0.00
28	19.49	13.90	0.00
29	39.34	42.64	16.67
30	38.90	42.41	
31	20.24	22.89	
32	43.90	42.09	4.17
33	14.30	13.22	8.44
34	24.87	23.45	6.85
35	4.51	5.29	2.85
36	28.13	24.56	
37	68.11	61.67	
38	51.79	54.13	
39	59.65	64.19	
40	83.58	65.28	
41	18.34	13.45	
42	8.03	10.69	
43	49.64	28.78	5.27
44	62.41	51.74	
45	22.34	12.37	
46	5.69	9.09	
47	20.00	17.02	0.00
48	47.05	44.89	
49	2.12	1.60	
50	31.19	34.89	
51	6.73	5.93	
52	28.91	34.66	
53	8.54	8.50	3.30
54	50.48	60.56	
55	12.72	11.05	7.75
56	28.14	27.22	12.23
57	37.39	32.12	
58	37.98	31.05	0.00
59	36.26	51.17	
60	22.56	27.87	5.32
61	28.90	44.36	
62	16.00	15.98	14.46
63	5.97	17.10	
64	17.40	17.61	7.38
65	14.28	15.88	5.78
66	8.80	10.30	
67	46.00	49.34	28.97
68	0.79	0.97	
69	15.53	12.24	0.73
70	29.56	41.62	
71	10.79	11.25	
72	37.61	51.02	
73	21.61	29.19	
74	24.86	33.77	
75	18.46	20.99	
76	42.89	46.64	42.96
77	42.32	54.07	
78	24.09	24.08	0.58
79	83.44	84.80	29.46
80	12.51	11.59	
81	14.12	10.71	1.30
82	37.00	54.50	
83	47.58	37.95	
84	2.35	2.98	0.00
85	12.98	12.60	11.45
86	15.53	21.01	
87	39.18	42.70	8.17

## Data Availability

The data presented in this study are available on request from the corresponding author. The data are not publicly available due to privacy reasons.
